# miR-125b Promotes Early Germ Layer Specification through Lin28/let-7d and Preferential Differentiation of Mesoderm in Human Embryonic Stem Cells

**DOI:** 10.1371/journal.pone.0036121

**Published:** 2012-04-24

**Authors:** Sharon S. Y. Wong, Carissa Ritner, Sweta Ramachandran, Julian Aurigui, Cameron Pitt, Piyanka Chandra, Vivian B. Ling, Odessa Yabut, Harold S. Bernstein

**Affiliations:** 1 Cardiovascular Research Institute, University of California San Francisco, San Francisco, California, United States of America; 2 Department of Pediatrics, University of California San Francisco, San Francisco, California, United States of America; 3 Graduate Program in Biomedical Sciences, University of California San Francisco, San Francisco, California, United States of America; 4 Eli and Edythe Broad Center of Regeneration Medicine and Stem Cell Research, University of California San Francisco, San Francisco, California, United States of America; University of Bristol, United Kingdom

## Abstract

Unlike other essential organs, the heart does not undergo tissue repair following injury. Human embryonic stem cells (hESCs) grow indefinitely in culture while maintaining the ability to differentiate into many tissues of the body. As such, they provide a unique opportunity to explore the mechanisms that control human tissue development, as well as treat diseases characterized by tissue loss, including heart failure. MicroRNAs are small, non-coding RNAs that are known to play critical roles in the regulation of gene expression. We profiled the expression of microRNAs during hESC differentiation into myocardial precursors and cardiomyocytes (CMs), and determined clusters of human microRNAs that are specifically regulated during this process. We determined that miR-125b overexpression results in upregulation of the early cardiac transcription factors, GATA4 and Nkx2-5, and accelerated progression of hESC-derived myocardial precursors to an embryonic CM phenotype. We used an in silico approach to identify Lin28 as a target of miR-125b, and validated this interaction using miR-125b knockdown. Anti-miR-125b inhibitor experiments also showed that miR-125b controls the expression of miRNA let-7d, likely through the negative regulatory effects of Lin28 on let-7. We then determined that miR-125b overexpression inhibits the expression of Nanog and Oct4 and promotes the onset of Brachyury expression, suggesting that miR-125b controls the early events of human CM differentiation by inhibiting hESC pluripotency and promoting mesodermal differentiation. These studies identified miR-125b as an important regulator of hESC differentiation in general, and the development of hESC-derived mesoderm and cardiac muscle in particular. Manipulation of miR-125b-mediated pathways may provide a novel approach to directing the differentiation of hESC-derived CMs for cell therapy applications.

## Introduction

Over five million people in the United States suffer with heart failure [1] because unlike other essential organs, the heart does not undergo tissue repair following injury [2]. Human embryonic stem cells (hESCs) grow indefinitely in culture while maintaining the ability to differentiate into many tissues of the body. As such, they provide a unique opportunity to elucidate the mechanisms that control human tissue development, as well as treat diseases characterized by tissue loss, including heart failure. We previously reported the identification of a human myocardial precursor derived from hESCs that gives rise to atrial, ventricular, and specialized conduction cardiomyocytes (CMs) [3]. The identification and isolation of this precursor now allows us to investigate further the regulatory events associated with early CM specification from hESCs.

MicroRNAs (miRNAs/miRs) are small, non-coding RNAs that were first identified as developmental mediators in C. elegans [4], [5], leading to the recognition that miRNAs play critical roles in the regulation of gene expression. miRNAs primarily support post-transcriptional gene silencing by targeting specific mRNA transcripts for degradation or by inhibiting their translation [6]. miRNAs are thought to control the expression/translation of >30% of all coding genes, and influence numerous biological processes, including stem cell pluripotency [6], lineage specification [7], tissue differentiation [8] and disease [9]. miRNAs are known to regulate genes and pathways important in normal development, and have been shown to promote tissue differentiation [6], [8].

Studies of Dicer mutants in zebrafish and mice have specifically implicated miRNA activity in early cardiac development. Zebrafish lacking Dicer show edematous cardiac tubes [10] suggesting poor function, and cardiac-specific deletion of Dicer in mice causes embryonic lethality at E12.5 with evidence of low-output heart failure [11]. In collaboration with colleagues, we have shown that miR-1 and -133 direct mesoderm formation, and that miR-1 directs subsequent cardiac differentiation, in hESCs by targeting the Notch ligand Delta-like-1 [7].

To identify miRNAs that support CM differentiation from hESCs, we profiled the expression of miRNAs during hESC differentiation into myocardial precursors and CMs in culture, and determined clusters of human miRNAs that appear to be specifically regulated during this process. We investigated further the role of miR-125b, and identified the Lin28/let-7 axis as the target pathway through which it functions during mesoderm and CM specification.

## Results

### miRNA expression is regulated during human CM differentiation

**Figure 1:**
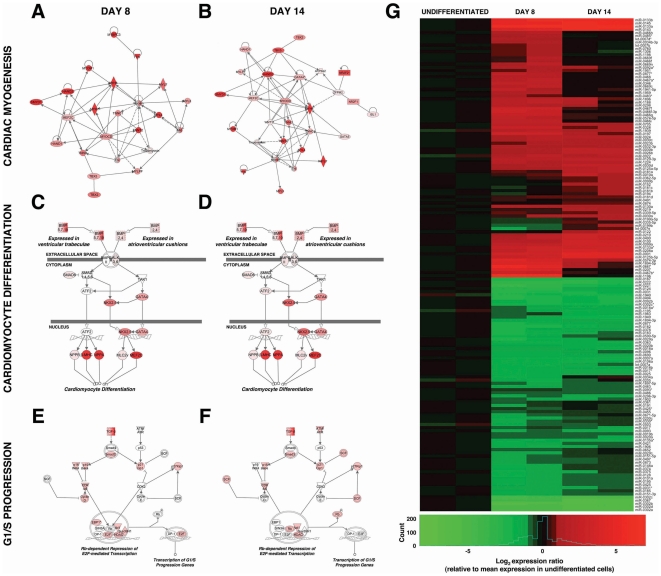
Differentiation of an αMHC-GFP reporter hESC line recapitulates cardiac myogenesis and identifies developmentally regulated miRNAs. mRNA expression profiles of hESC-derived, αMHC-GFP^+^ myocardial precursors sorted at days 8 (**A, C, E**) and 14 (**B, D, F**) of differentiation demonstrated upregulated expression (red) of genes involved in cardiac myogenesis (**A, B**), cellular differentiation into CMs (**C, D**), and withdrawal from the cell cycle (**E, F**). Abl1, c-abl non-receptor tyrosine kinase; ALK, BMP receptor; ATF2, activating transcription factor 2; ATM/ATR, ; ATP2A2, cardiac calcium transporting ATPase; BMP, bone morphogenic protein; BMPR, BMP receptor; CDK, cyclin-dependent kinase; DP-1, transcription factor Dp-1; E2F, E2F transcription factor; EBP1, ErbB3 binding protein; GATA, GATA binding protein; HAND, heart and neural crest derivatives expressed factor; HDAC, histone deacetyltransferase; ISL1, islet-1; MEF2C, myocyte enhancer factor 2C; MLC2v, ventricular myosin light chain 2; MYH6, α-myosin heavy chain; MYL3, ventricular myosin light chain 3; MYL4, atrial myosin light chain 4; MYLPF, fast skeletal myosin light chain; MYOCD, myocardin; MYOM1, myomesin 1; NKX2.5, NK2 transcription factor related locus 5; NPPA, natriuretic peptide A; NPPB, natriuretic peptide B; NR2F, nuclear receptor subfamily 2, group F; p15INK4, cyclin-dependent kinase inhibitor 2B; p16INK4, cyclin-dependent kinase inhibitor 2A; p21Cip1, cyclin-dependent kinase inhibitor 1A; p27Kip1, cyclin-dependent kinase inhibitor 1B; p53, 53 kD tumor protein ; Rb, retinoblastoma protein; SCF, Skp-Cullin-F-box containing E3 ubiquitin ligase complex; SIN3A, SIN3 transcription factor homolog A; SMAD, SMA/mothers against decapentaplegic; SMYD1, SET and MYND domain containing protein; Suv39H1, suppressor of variegation 3–9 homolog 1; TAK1, mitogen-activated protein kinase kinase kinase 7; TBX, T-box transcription factor; TGF-β, transforming growth factor-β; TnI, cardiac troponin I; TNNC1, troponin C type 1; TNNI1, troponin I type 1; TNNT2, cardiac troponin T ; TTN, titin; WNT11, wingless-type MMTV integration site family member 11; ZFPM2, zing finger protein multitype 2; β-MHC, β-myosin heavy chain. **G**) Relative miRNA expression in αMHC-GFP^+^ hESCs sorted at days 8 and 14 of differentiation (two samples each), compared to undifferentiated αMHC-GFP reporter hESCs. Heat map shows log_2_-fold change (sample intensity/mean intensity for undifferentiated group) for the 156 miRNAs that had statistically significant differences (false discovery rate (FDR)<0.05) of at least 2-fold in any pairwise comparison between the three groups.

To investigate the role of miRNAs in human CM differentiation, we performed expression profiling of miRNAs expressed by differentiating, α-myosin heavy chain (αMHC)-enhanced green fluorescent protein (GFP)^+^ myocardial precursors isolated from a previously decribed myocardial reporter hESC line [3]. We compared expression profiles of undifferentiated hESCs to αMHC-GFP^+^ cells sorted at days 8 and 14 of differentiation. These time points coincide with induction of the early cardiac transcription factor, Nkx2-5, and the definitive CM structural protein, cardiac troponin I (cTnI), respectively [3]. To validate the analysis, we evaluated expression of genes specifically involved in cardiac myogenesis, CM differentiation, and cell cycle withdrawal (**Figure 1A–F**). We observed upregulation of mRNAs involved in cardiac myogenesis (Mef2C, HAND1, HAND2, TBX2, TBX5, MLC2a, MLC2v, cTnI; **Figure 1A,B**), signaling pathways that effect ventricular trabeculation and atrioventricular cushion formation (BMP5/7/10 and BMP2/4, Nkx2-5, GATA4, Mef2C, βMHC; **Figure 1C,D**), and cell cycle withdrawal coincident with differentiation (p16^INK4^, p15^INK4B^, p21^CIP1^, p27^KIP1^, SCF; **Figure 1E,F**) over the 14 day course of differentiation. We confirmed the regulated expression of a subset of these genes by quantitative real-time PCR (qPCR; **Figure S1**).

We then examined the expression patterns of >14,500 unique miRNA sequences representing >900 human miRNAs from sorted αMHC-GFP^+^ cells at days 8 and 14 of differentiation compared to undifferentiated hESCs. This identified 95 miRNAs exhibiting ≥2-fold change in expression (False Discovery Rate (FDR)<0.05) at day 8 and 67 miRNAs exhibiting ≥2-fold change in expression (FDR<0.05) at day 14. In addition, a heat map of log_2_-fold change of all arrays relative to the average of the undifferentiated hESC group for genes that were differentially expressed in any of the comparisons, clustered using Euclidean distance with average linkage, showed appropriate clustering according to time point groups (**Figure 1G**). This confirmed that the arrays were able to detect miRNA expression differences due to biological differences between the time points examined. Among those miRNAs demonstrating the most significant changes in expression over the course of CM differentiation (**Table 1**), several have previously been shown to play a role in early cardiac specification, including miR-1 and miR-133 [7]. Others, such as let-7, have been implicated more generally in the promotion of terminal differentiation in vertebrates [12]. Of particular interest, miR-125b previously has been observed as an important effector of mesenchymal differentiation, including skeletal muscle and bone [13], [14]. In addition, it has been implicated in the suppression of cellular proliferation in various cancers [15]–[17].

**Table 1:** miRNAs exhibiting significant change in expression during CM differentiation.

*miRNAs with fold change >2 (FDR<0.05)*						
	*Day 8 vs. Undifferentiated*			*Day 14 vs. Undifferentiated*		
	Fold Change	FDR	AdjP	Fold Change	FDR	AdjP
let-7d*	2.5	0.002	0.050	0.4	0.015	0.706
miR-1	11.1	0.002	0.084	16.9	<0.001	0.020
miR-23b	2.2	0.008	0.417	3.0	0.002	0.044
miR-30d	2.7	0.002	0.074	5.4	<0.001	0.002
miR-125a-5p	2.2	0.004	0.180	4.2	<0.001	0.003
miR-125b	7.8	0.001	0.032	4.2	<0.001	0.003
miR-129-3p	3.7	<0.001	0.009	4.4	<0.001	0.002
miR-133a	75.4	<0.001	<0.001	72.5	0.000	<0.001
miR-133b	48.8	<0.001	<0.001	56.2	<0.001	<0.001
miR-143	20.5	0.002	0.063	43.8	<0.001	0.010
miR-145	43.1	<0.001	<0.001	59.7	0.000	<0.001
miR-197	8.3	<0.001	0.003	2.3	0.010	0.438
miR-210	3.3	0.003	0.148	8.6	<0.001	0.003
miR-328	6.5	<0.001	0.024	2.4	0.019	0.908
miR-490-3p	3.6	<0.001	0.024	7.7	<0.001	0.001
miR-532-3p	2.5	0.002	0.071	2.6	0.002	0.041
miR-574-3p	17.6	<0.001	0.001	3.4	0.004	0.142

*miRNAs with fold change <0.5 (FDR<0.05)*						
	*Day 8 vs. Undifferentiated*			*Day 14 vs. Undifferentiated*		
	Fold Change	FDR	AdjP	Fold Change	FDR	AdjP
miR-7	0.30	<0.001	0.016	0.31	<0.001	0.011
miR-124	0.13	<0.001	<0.001	0.36	0.001	0.039
miR-183	0.24	0.001	0.031	0.46	0.011	0.525
miR-187	0.16	<0.001	0.002	0.15	<0.001	0.001
miR-221	0.08	<0.001	0.004	0.16	0.001	0.013
miR-222	0.09	<0.001	<0.001	0.15	<0.001	0.003
miR-302a	0.02	<0.001	0.006	0.01	<0.001	<0.001
miR-302b	0.02	<0.001	<0.001	0.01	<0.001	<0.001
miR-302d	0.03	<0.001	0.012	0.01	<0.001	0.002
miR-367	0.05	0.002	0.084	0.01	<0.001	0.003
miR-378	0.27	0.001	0.028	0.45	0.008	0.299
miR-494	0.16	0.001	0.039	0.25	0.004	0.129

### miR-125b expression is developmentally regulated during human CM differentiation

**Figure 2:**
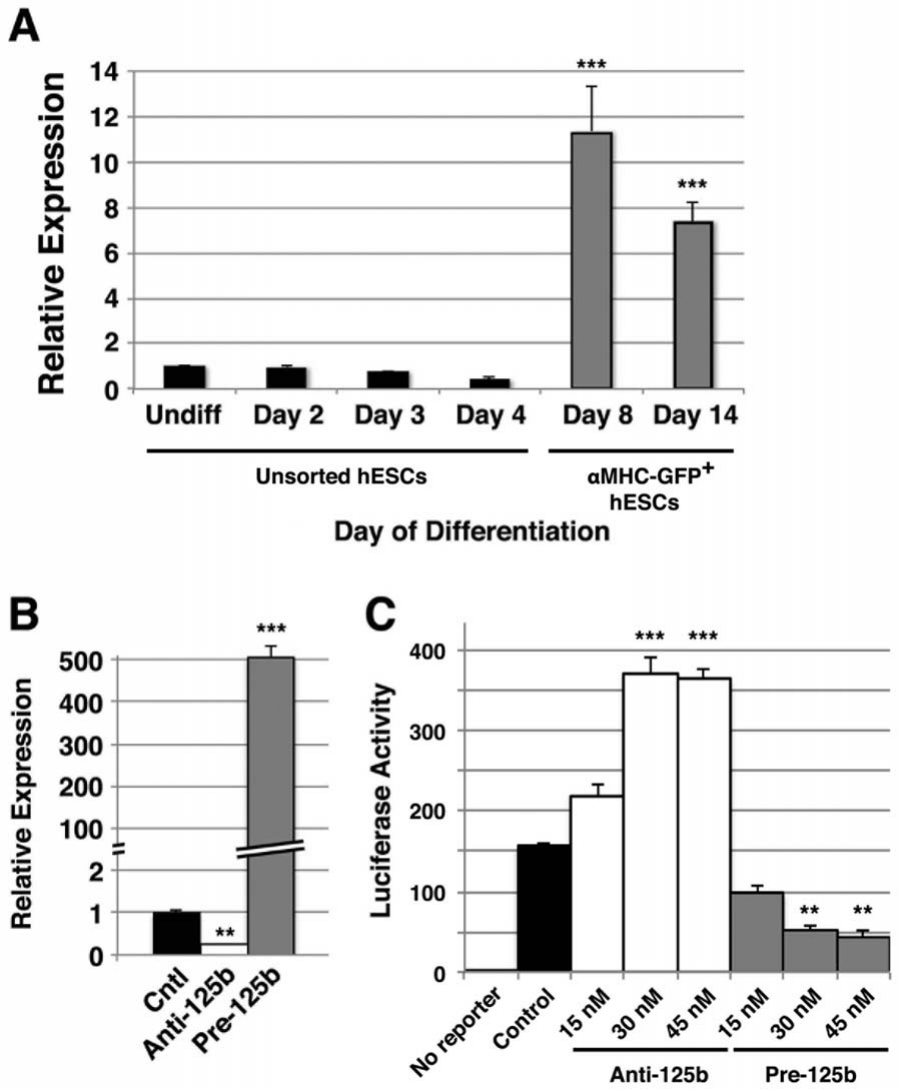
miR-125b expression increases with differentiation in hESCs. **A**) Relative expression of endogenous miR-125b in undifferentiated hESCs (Undiff) and hESCs grown in differentiation medium for 2, 3, and 4 days, or sorted αMHC-GFP^+^ hESCs differentiated for 8 and 14 days (as for **Figure 1**) was assessed by qPCR. A significant increase in miR-125b expression was observed in sorted αMHC-GFP^+^ hESCs at day 8 and sustained through day 14 of differentiation. Data shown are mean±s.e.m. (N = 3). ***, p<0.001. **B**) Relative expression of miR-125b in undifferentiated hESC cultures transfected with 30 nM anti-miR-125b inhibitor (anti-125b) or pre-miR-125b (pre-125b), as analyzed by qPCR, showed appropriate down- or up-regulation of miR-125b expression, respectively, compared to untransfected control (Ctl) hESCs. **C**) Relative binding and activity of miR-125b in undifferentiated hESC cultures transfected with anti-125b and pre-125b, as assessed by luciferase reporter activity, demonstrated appropriate up- and down-regulation of luciferase activity in a dose-dependent manner, respectively, compared to hESCs transfected with luciferase reporter alone (Ctl). Data shown are mean±s.e.m. (N = 3). **, p<0.01; ***, p<0.001.

To validate the expression of miR-125b over the course of hESC differentiation, we analyzed the expression of miR-125b by qPCR in undifferentiated H9 hESCs compared to H9 hESCs cultured under differentiation conditions for 2, 3, and 4 days, and H9-derived αMHC-GFP^+^ hESCs at 8 and 14 days (**Figure 2A**). This demonstrated that miR-125b expression increased >10-fold by day 8 of hESC differentiation into CM precursors compared to undifferentiated hESCs (p<0.001), consistent with expression profiling analysis (**Table 1**). To confirm that miR-125b expression was consistent across hESC lines, we analyzed its expression in the unrelated H7 hESC line (**Figure S2**). The expression of miR-125b was similar between differentiating H7 and H9 hESCs.

### miR-125b promotes CM differentiation from hESCs

To determine the effects of miR-125b on CM differentiation specifically, we transfected proliferating hESCs with pre-miR-125b and anti-miR-125b inhibitor to achieve overexpression and knockdown of miR-125b, respectively (**Figure 2B, C**). Expression analysis of miR-125b by qPCR demonstrated a 4.3-fold decrease in miR-125b expression with anti-miR compared to control (0.23±0.03 vs. 1.00±0.09; p<0.01), and a >500-fold increase in miR-125b expression with pre-miR compared to control (541.44±19.29 vs. 1.00±0.09; p<0.001) (**Figure 2B**). To evaluate miR-125b binding and activity with anti- and pre-miR transfection, we co-transfected hESCs with a luciferase reporter containing the predicted miR-125b binding site (**Figure 2C**). This demonstrated a dose-dependent decrease in luciferase activity with expression of pre-miR-125b compared to control cells (minimum relative light units (RLU) 52.78±9.63 vs. 155.50±12.00; p<0.01), and a dose-responsive increase in luciferase activity with expression of anti-miR-125b (maximum RLU 373.12±23.55 vs. 155.50±12.00; p<0.01) (**Figure 2C**). This analysis confirmed appropriate manipulation of miR-125b expression, binding, and activity in culture with pre- and anti-miR-125b constructs.

**Figure 3:**
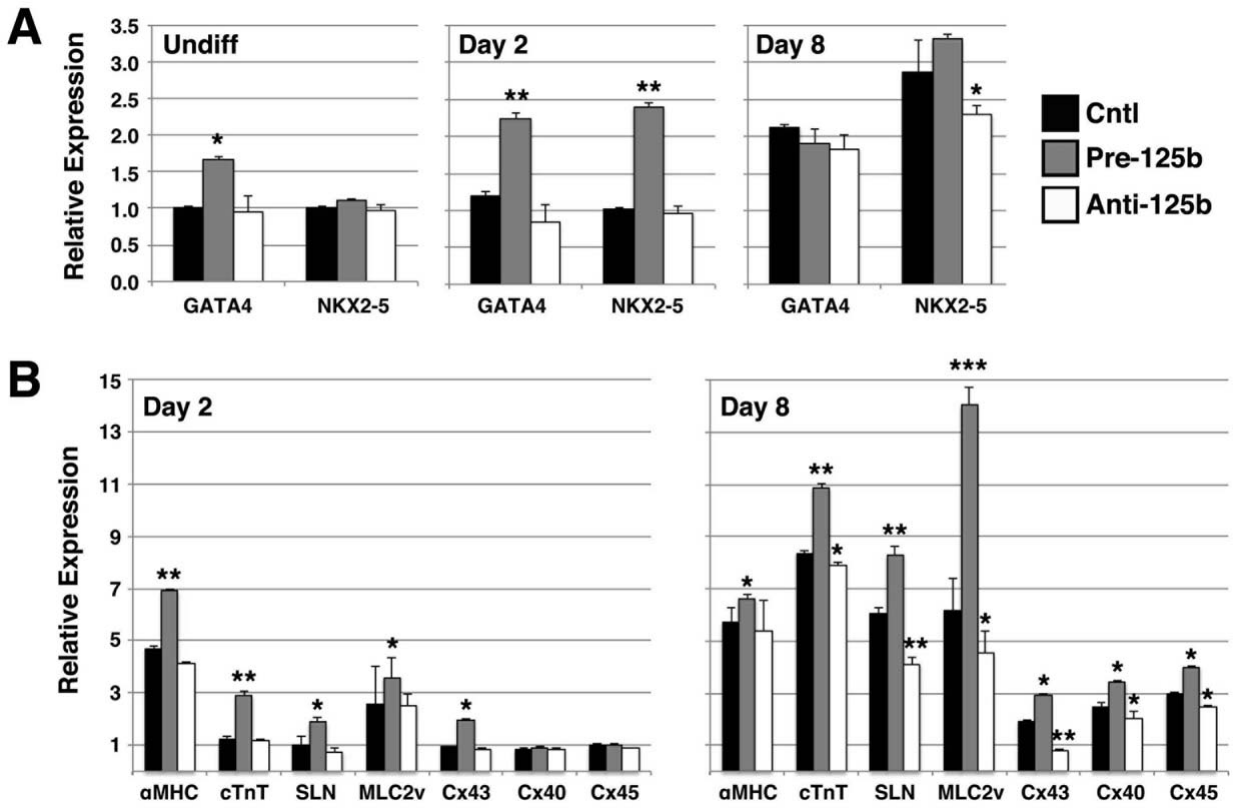
miR-125b promotes the expression of cardiac-specific genes during hESC differentiation. hESCs were transfected with pre-miR-125b (pre-125b) or anti-miR-125b inhibitor (anti-125b), cultured in differentiation medium for 2 or 8 days, and analyzed for expression of cardiac-specific mRNAs by qPCR. **A**) Overexpression of pre-125b induced premature expression of the early cardiac transcription factor, GATA4, in undifferentiated hESCs (Undiff), and promoted the early expression of both GATA4 and Nkx2-5 in hESCs cultured in differentiation medium for only 2 days, before the usually observed expression of these transcription factors during CM differentiation. Anti-125b suppressed the expression of Nkx2-5 at day 8 of differentiation, but had little effect on GATA4 at this time point. Data shown are mean±s.e.m. (N = 3). *, p<0.05; **, p<0.01. **B**) Overexpression of pre-125b resulted in the early expression of CM structural genes at day 2, before they are usually observed, while expression of anti-125b inhibited the expression of these genes at day 8, when they begin to be expressed in differentiating hESCs. αMHC, α-myosin heavy chain; cTnT, cardiac troponin T; SLN, sarcolipin; MLC2v, ventricular myosin light chain 2; Cx, connexin. Data shown are mean±s.e.m. (N = 3). *, p<0.05; **, p<0.01; ***, p<0.001.

We then examined the effects of miR-125b overexpression and knockdown on the expression of CM-specific genes over the course of hESC differentiation (**Figure 3**). Expression analysis of the early cardiac transcription factor, GATA4, with miR-125b overexpression showed premature upregulation of GATA4 expression in undifferentiated hESCs (1.67±0.03 vs. 1.00±0.03; p<0.05) as well as hESCs grown in differentiation medium for 2 days (2.24±0.08 vs. 1.20±0.05; p<0.01) before GATA4 expression is normally observed [3] (**Figure 3A**). Appropriate expression of GATA4 in hESCs differentiated for 8 days was unaffected by overexpression of miR-125b (1.91±0.19 vs. 2.13±0.04; p>0.05). Interestingly, overexpression of miR-125b in undifferentiated hESCs did not effect Nkx2-5 expression (1.11±0.03 vs. 1.00±0.04; p>0.05); however, it did result in premature upregulation of Nkx2-5 expression in hESCs cultured in differentiation media for 2 days (2.40±0.05 vs. 1.01±0.02; p<0.01), before Nkx2-5 expression is normally seen [3] (**Figure 3A**). Knockdown of miR-125b had the opposite effects on GATA4 and Nkx2-5 expression over the course of hESC differentiation. These data suggested that miR-125b promotes the onset of CM differentiation from hESCs.

Expression of genes that are active later during CM differentiation was similarly affected by miR-125b overexpression and knockdown (**Figure 3B**). Normally, expression of genes associated with embryonic ventricle (αMHC, cardiac troponin T (cTnT), ventricular myosin light chain-2), atrium (αMHC, cTnT, sarcolipin), and conduction tissue (connexin 43) is not observed in hESC-derived CMs until day 8 of differentiation [3]. However, premature expression of all of these genes was observed in hESCs transfected with pre-miR-125b and cultured in differentiation medium for only 2 days (**Figure 3B**). Similarly, inhibition of these genes was seen in anti-miR-125b-transfected hESCs cultured in differentiation medium for 8 days (**Figure 3B**). Of note, the expression of connexins 40 and 45, which are expressed later during development of the conduction system, were not affected by miR-125b overexpression until day 8, after the onset of Cx43 expression (**Figure 3B**). These data support a persistent role for miR-125b that extends into the later stages of CM development during hESC differentiation.

### In silico prediction of miR-125b targets

**Figure 4:**
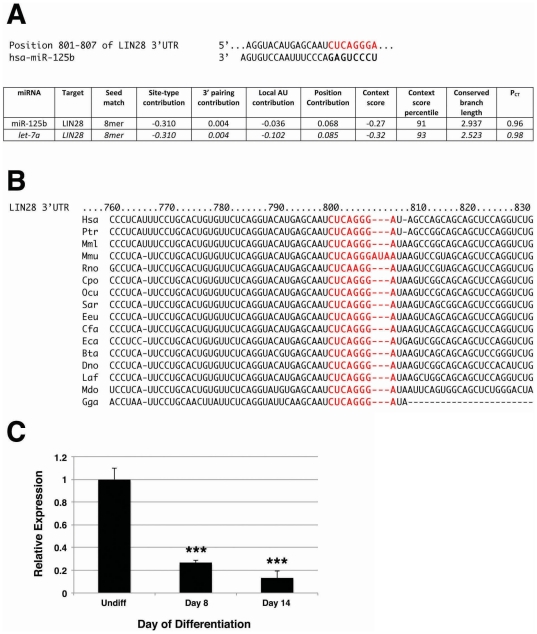
Lin28 is a predicted target of miR-125b in differentiating hESCs. **A**) In silico analysis of potential miR-125b targets using Target Scan Human identified Lin28 as a potential target. Seed match refers to the match between a sequence in the 3′ untranslated region (UTR) of the putative target gene and positions 2–8 of the mature miRNA; site-type contribution reflects the average contribution of each site identified; 3′ pairing contribution describes the impact of miRNA-target complementarity outside the seed region; local AU contribution is a measure of the target transcript AU content 30 nucleotides upstream and downstream of the predicted site; position contribution reflects the distance to the nearest end of the target's UTR. For each of these criteria, a more negative score indicates a more favorable site. The context score is the sum of the contributions of site-type, 3′ pairing, local AU, and position criteria. Conserved branch length is the sum of phylogenetic branch lengths between species that contain the site. The probability of conserved targeting (P_CT_) reflects the Bayesian estimate of the probability that a site is evolutionarily conserved through selective advantage, rather than by chance. These criteria applied to miR-125b/Lin28 were similar to those for the established let-7a/Lin28 interaction. **B**) Conservation among mammals of the miR-125b binding site in the 3′ UTR of Lin28. Bta, cow; Cfa, dog; Cpo, guinea pig; Dno, armadillo; Eca, horse; Eeu, hedgehog; Gga, chicken; Hsa, human; Laf, elephant; Mdo, opossum; Mml, rhesus; Mmu, mouse; Ocu, rabbit; Ptr, chimpanzee; Rno, rat; Sar, shrew. **C**) Undifferentiated αMHC-GFP reporter hESCs (Undiff) and αMHC-GFP^+^ CMs sorted from reporter hESCs grown in differentiation medium for 8 or 14 days were evaluated for Lin28 expression by qPCR. Endogenous expression of Lin28 mRNA declined with CM differentiation. Data shown are mean±s.e.m. (N = 3). ***, p<0.001.

We used Target Scan Human (Release 5.2; MIT) to predict potential targets of miR-125b. Lin28 was among those targets identified with the highest probabilities of conserved targeting (P_CT_; [18]), based on seed match [19], site-type contribution [20], 3′ pairing contribution outside the seed region [20], overall context score [20], and conserved branch length score [18] (**Figure 4A**). These criteria compared favorably with those for the previously validated interaction between let-7a and Lin28 [21] (**Figure 4A**). The putative miR-125b binding site in the Lin28 3′ untranslated sequence was also highly conserved among mammals (**Figure 4B**). In addition, Lin28 has been identified as a target of miR-125b during neuronal differentiation of mouse P19 embryonal carcinoma cells [22], and more recently, miR-125b has been shown to target Lin28 during mouse embryoid body formation [23]. Taken together, this analysis strongly supported the identification of Lin28, recognized for its role in the maintenance of stem cell pluripotency [24], as a potential target for human miR-125b.

### miR-125b targets the Lin28/let-7 axis during hESC differentiation

**Figure 5:**
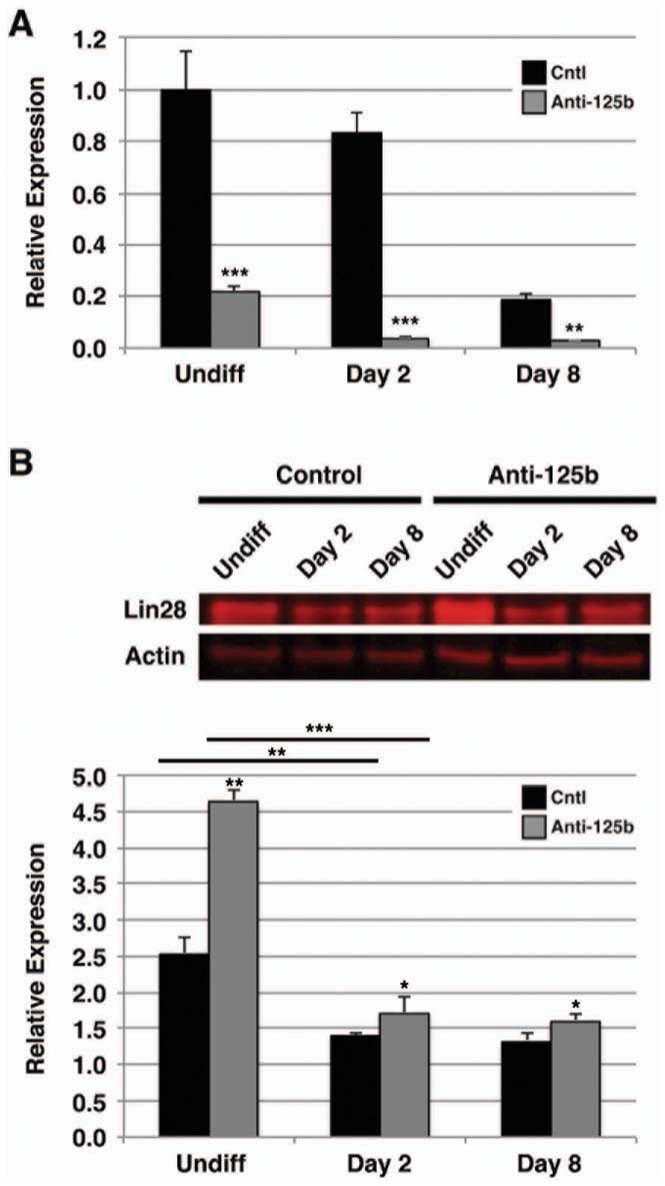
Lin28 expression is controlled by miR-125b in differentiating hESCs. Untransfected hESCs (Cntl) or hESCs transfected with anti-miR-125b inhibitor (anti-125b) were cultured in differentiation medium for 2 or 8 days. **A**) Cells were analyzed for expression of miR-125b by qPCR. Appropriate downregulation of mature miR-125b was seen in both undifferentiated and differentiating hESCs. Data shown are mean±s.e.m. (N = 3). **, p<0.01; ***, p<0.001. **B**) Cell lysates were assayed for Lin28 by immunoblot analysis compared to undifferentiated cells (Undiff). Lin28 protein expression was noticeably decreased in hESCs differentiated for 2 or 8 days compared to undifferentiated cells, while transfection with anti-125b induced Lin28 expression in undifferentiated as well as differentiating cells (TOP). Actin was used as a loading control. Representative results are shown. Quantitation of fluorescent signals is shown (BOTTOM). Data shown are mean±s.e.m. (N = 3). *, p<0.05; **, p<0.01; ***, p<0.001.

To determine whether Lin28 expression inversely parallels miR-125b expression during CM differentiation, we analyzed Lin28 transcription by qPCR in undifferentiated hESCs compared to αMHC-GFP^+^ myocardial precursors and CMs sorted from cultures grown in differentiation medium for 8 and 14 days, respectively (**Figure 4C**). We observed a significant decrease in Lin28 mRNA over time (Day 8: 0.27±0.02 vs. 1.00±0.1, p<0.001; Day 14: 0.13±0.06 vs. 1.00±0.1, p<0.001), as would be predicted by negative post-transcriptional regulation of Lin28 by miR-125b. To determine whether the change in Lin28 expression with differentiation was mediated by miR-125b, we knocked down miR-125b in differentiating hESCs (**Figure 5A**), and assayed Lin28 protein expression (**Figure 5B**). In undifferentiated cells, expression of anti-miR-125b lead to an increase in Lin28 expression. As Lin28 expression decreased with hESC differentiation, transfection with anti-miR-125b had a similar effect compared to untransfected cells, although to a lesser extent. This demonstrated that the effects of miR-125b on early hESC differentiation likely occur through inhibition of Lin28.

**Figure 6:**
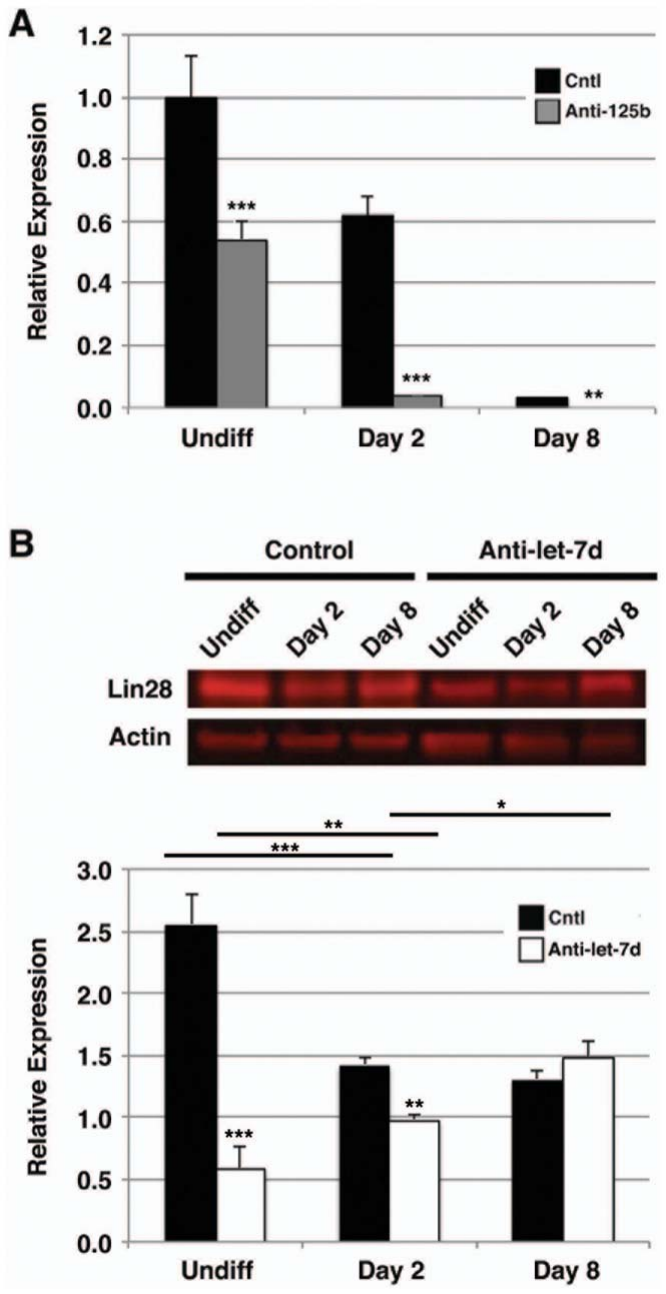
Lin28-mediated let-7d expression is regulated by miR-125b in differentiating hESCs. Untransfected hESCs (Cntl) or hESCs transfected with anti-miR-125b (anti-125b) or anti-let-7d (anti-let-7d) inhibitor were cultured in differentiation medium for 2 or 8 days. **A**) Cells were analyzed for expression of let-7d by qPCR. Inhibition of miR-125b downregulated expression of let-7d in both undifferentiated and differentiating hESCs. Data shown are mean±s.e.m. (N = 3). **, p<0.01; ***, p<0.001. **B**) Cell lysates were assayed for Lin28 by immunoblot analysis compared to undifferentiated cells (Undiff). Lin28 protein expression was noticeably decreased in undifferentiated hESCs transfected with anti-let-7d compared to untransfected undifferentiated cells. This effect was seen to a lesser extent in hESCs differentiated for 2 days. However, the effect of let-7d inhibition on Lin28 was lost by day 8 of differentiation (TOP). Actin was used as a loading control. Representative results are shown. Quantitation of fluorescent signals is shown (BOTTOM). Data shown are mean±s.e.m. (N = 3). *, p<0.05; **, p<0.01; ***, p<0.001.

Since Lin28 has been shown to exert its effects on pluripotency through binding to and inactivation of let-7, we examined the effect of miR-125b knockdown on let-7d and let-7d* expression in differentiating hESCs. We focused on let-7d and let-7d* because let-7d* demonstrated regulated expression during our initial expression profiling screen (**Table 1**). MiR-125b knockdown resulted in parallel downregulation of let-7d expression (**Figure 6A**). Interestingly, a similar effect on let-7d* was not observed (data not shown), suggesting that its expression during hESC differentiation is regulated independent of miR-125b. Since let-7 family members in mammals have been shown to inhibit Lin28 [25], suggesting that let-7 might participate in negative feedback to its negative regulator, we examined the effect of let-7d knockdown on Lin28 expression (**Fig. 6B**). Surprisingly, expression of anti-let-7d in differentiating hESCs resulted in downregulation of Lin28, suggesting that let-7d may, in fact, positively regulate its negative regulator, Lin28, during hESC differentiation.

### miR-125b promotes early events of cardiac mesoderm development by inhibiting embryonic stem cell pluripotency and promoting mesodermal differentiation

**Figure 7:**
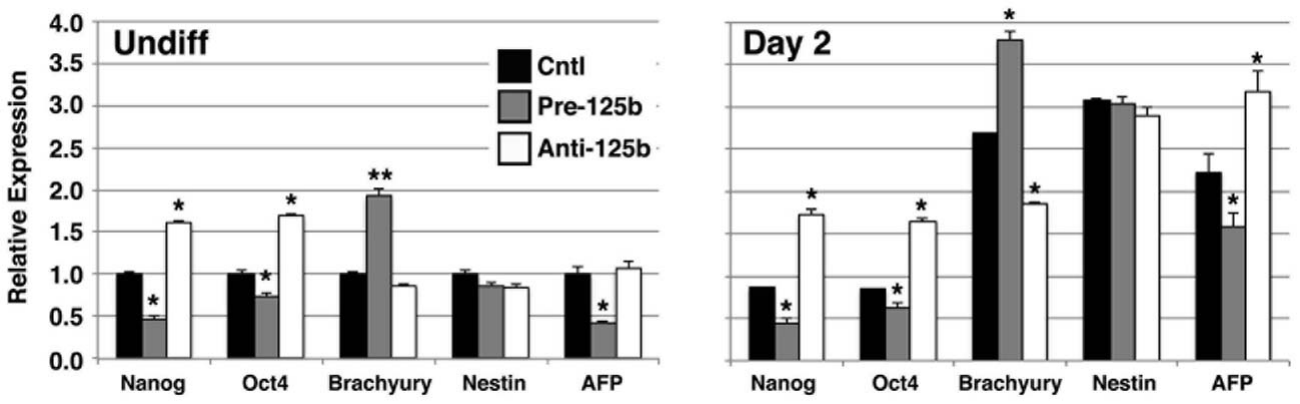
miR-125b inhibits the expression of pluripotency genes and promotes mesodermal development during hESC differentiation. hESCs were transfected with pre-miR-125b (pre-125b) or anti-miR-125b inhibitor (anti-125b), cultured in differentiation medium for 2 days, and analyzed for expression of pluripotency or early germ layer mRNAs by qPCR. Overexpression of pre-125b suppressed the expression of pluripotency markers, Nanog and Oct4, in undifferentiated hESCs and induced premature expression of the early mesodermal marker, Brachyury. Anti-125b promoted the expression of Nanog and Oct4 at day 2 of differentiation, and conversely inhibited the normal expression of Brachury at this time point. AFP, α-fetoprotein. Data shown are mean±s.e.m. (N = 3). *, p<0.05; **, p<0.01.

Since Lin28 regulates pathways controlling pluripotency and differentiation [24], we examined whether manipulation of miR-125b expression in undifferentiated and differentiating hESCs affected the expression of other pluripotency genes, i.e., Nanog and Oct4, as well as genes expressed early during development of mesoderm, ectoderm, and endoderm (i.e., Brachyury, Nestin, and α-fetoprotein, respectively) (**Figure 7**). In undifferentiated hESCs, overexpression of pre-miR-125b suppressed Nanog and Oct4 expression (Nanog: 0.47±0.04 vs. 1.00±0.03, p<0.05; Oct4: 0.74±0.04 vs. 1.00±0.04, p<0.05), and promoted the premature expression of Brachyury (1.93±0.08 vs. 1.00±0.03, p<0.01), compared to control cells. Conversely, expression of anti-miR-125b in hESCs grown in differentiation medium for 2 days (early differentiation) resulted in higher levels of Nanog and Oct4 expression (Nanog: 1.74±0.06 vs. 0.87±0.01, p<0.05; Oct4: 1.65±0.04 vs. 0.84±0.01, p<0.05), and suppression of Brachyury (1.86±0.01 vs. 2.68±0.01, p<0.05). Interestingly, Nestin, a marker of primitive ectoderm, did not appear to be affected by miR-125b expression, and the primitive endodermal marker, α-fetoprotein, showed expression patterns opposite of those for Brachyury with overexpression of pre-miR-125b (undifferentiated/pre-miR-125b: 0.43±0.03 vs. 1.00±0.09, p<0.05; differentiated/anti-miR-125b: 3.18±0.25 vs. 2.23±0.21, p<0.05). These data suggest that miR-125b promotes withdrawal from the pluripotency state, likely through its effects on Lin28, and that it also preferentially favors the development of mesoderm, including cardiac muscle, over endoderm.

## Discussion

The small regulatory RNA, miR-125b, has previously been shown to function during the differentiation of tissues from mesodermal precursors, including osteoblasts from mesenchymal stem cells [14] and skeletal muscle from C2C12 myoblasts [13]. In addition, recent studies have implicated miR-125b in the early commitment of stem cells to skin elements [26]. However, the specific targets through which miR-125b mediates these effects are not completely known. We now demonstrate a role for miR-125b in promoting the differentiation of mesoderm including CMs from hESCs, and that this likely occurs in part through miR-125b targeting of the pluripotency factor, Lin28.

Lin28 has been identified as a marker of undifferentiated embryonic stem cells [27], and has been used to enhance the efficiency of the formation of induced pluripotent stem cells from fibroblasts [28]. A recent study of Lin28 in hESCs suggested a role in hESC fate determination, specifically the switch from self-renewal to differentiation, and also implicated Lin28 in promoting the formation of specific tissues [29]. Our experiments underscore the role of Lin28 in early hESC differentiation, and trace its regulation to miR-125b.

Members of the let-7 miRNA family in vertebrates are believed to play a role in cell differentiation based on temporal expression during development [30] and low levels of expression in undifferentiated tumors [31]. Recent studies have elucidated the mechanisms by which let-7 biogenesis and activity are inhibited by Lin28 [32], [33]. Since a let-7/Lin28 negative feedback loop has also been shown in vertebrates [25], we were surprised to observe that let-7d appears to positively regulate Lin28 expression. Although further investigation of this observation is warranted, this positive feedback loop may somehow titrate the tempo of differentiation and withdrawal from the pluripotent state. The effect of let-7d on Lin28 also may be one of several signals converging on the Lin28 axis, with the balance of these inputs determining hESC fate.

While our experiments indicate that miR-125b plays a regulatory role in the early stages of hESC differentiation, likely through targeting Lin28, it also appears to induce the formation of mesoderm, and cardiac mesoderm in particular. This, however, is not likely to involve Lin28, as Lin28 expression decreases dramatically with hESC differentiation, suggesting that other potential targets of miR-125b may be responsible. Target Scan identified 604 conserved targets for miR-125b, with >50 demonstrating an aggregate P_CT_ >0.95 (Table S1), and 49 with a total context score ≤−0.45 (Table S2). Some of these target gene products may participate in pathways that promote endoderm or ectoderm, or even non-cardiac mesoderm, and miR-125b may mediate its developmental preferences by negatively regulating these. Further investigation is warranted to elucidate these mechanisms.

In summary, using an αMHC-GFP reporter hESC line, we have identified miR-125b as an important regulator of hESC differentiation in general, and the development of hESC-derived mesoderm including cardiac muscle. Further investigation of miR-125b-mediated pathways will provide important insight into the regulation of human myocardial development, and provide a novel approach to directing the differentiation of hESC-derived CMs for cell therapy applications.

## Materials and Methods

### hESC culture and differentiation

All work with hESCs was done with the approval of the UCSF Stem Cell Research Oversight Committee. The αMHC-GFP myocardial reporter [3], H9 (WA09; WiCell) and H7 (WA07; WiCell) hESC lines were maintained on irradiated mouse embryonic fibroblast feeder cells [3] in a medium comprised of Knockout DMEM (Invitrogen) supplemented with 20% Knockout Serum Replacement (Invitrogen), 2 mM glutamine, 0.1 mM nonessential amino acids, 0.1 mM β-mercaptoethanol and 15 ng/ml recombinant human FGF-basic (R&D Systems). Differentiation was initiated by human embryoid body (hEB) formation in suspension as previously described [3], [34]. Briefly, colonies of hESCs were dissociated into clusters by exposure to Collagenase IV (Sigma-Aldrich), then allowed to differentiate in a medium comprised of Knockout DMEM (Invitrogen) supplemented with 20% Defined Fetal Bovine Serum (Hyclone), 2 mM glutamine, 0.1 mM non-essential amino acids, and 0.1 mM β-mercaptoethanol. After 4 days in suspension, hEBs were attached to gelatin-coated 12-well culture plates and allowed to differentiate for an additional 14 days. For expression profiling experiments, hEBs were dissociated with TrypLE Express (Invitrogen) to generate single cell suspensions, stained with propidium iodide to distinguish between live and dead cells, and sorted on the basis of GFP expression using a FACSAria (Becton Dickinson) with standard filter sets using previously described methods [3].

### mRNA expression profiling

Sample preparation, labeling, and array hybridizations were performed as previously described [3], according to standard protocols from the UCSF Shared Microarray Core Facilities and Agilent Technologies (http://www.arrays.ucsf.edu and http://www.agilent.com). Total RNA quality was assessed using a Pico Chip on an Agilent 2100 Bioanalyzer (Agilent Technologies). RNA was amplified using the Sigma whole transcriptome amplification kit following the manufacturer's protocol (Sigma-Aldrich), and subsequent Cy3-CTP labeling was performed using the NimbleGen one-color labeling kit (Roche-NimbleGen). The size distribution and quantity of the amplified product was assessed using an Agilent 2100 Bioanalyzer and a Nanodrop ND-8000 (Nanodrop Technologies); the labeled DNA was assessed using the Nandrop 8000, and equal amounts of Cy3 labeled target were hybridized to Agilent human whole genome 4x44K Ink-jet arrays. Hybridizations were performed for 14 hrs, according to the manufacturers protocol. Arrays were scanned using an Agilent microarray scanner and raw signal intensities were extracted with Feature Extraction v.10.1 software (Agilent Technologies). Data were further analyzed using Ingenuity Pathways Analysis (Ingenuity Systems) to identify biological pathways involved in CM differentiation. The false discovery rate (FDR) from the data set was used for canonical pathways analysis.

Data were normalized using the quantile normalization method [35]. No background subtraction was performed, and the median feature pixel intensity was used as the raw signal before normalization. A one-way ANOVA linear model was fit to the comparison to estimate the mean M values and calculated FDR for each gene for the comparison of interest. All procedures were carried out using functions in the R package *limma* in Bioconductor [36], [37]. All data comply with MIAME standards for microarray experiments [38], and have been deposited in Gene Expression Omnibus (http://www.ncbi.nlm.nih.gov/geo/; Accession Number GPL6480).

### miRNA expression profiling

Sample preparation, labeling, and array hybridizations were performed according to standard protocols from the UCSF Shared Microarray Core Facilities and Agilent Technologies (http://www.arrays.ucsf.edu and http://www.agilent.com). Total RNA quality was assessed using a Pico Chip on an Agilent 2100 Bioanalyzer (Agilent Technologies). RNA was labeled with Cy3-CTP using the miRCURY LNA microRNA power labeling kit (Exiqon), according to manufacturers protocol. Labeled RNA was hybridized to Agilent custom UCSF miRNA v3.4 multi-species 8x15K Ink-jet arrays (Agilent Technologies). Hybridizations were performed for 16 hrs, according to the manufacturers protocol (Agilent Technologies). Arrays were scanned using the Agilent microarray scanner (Agilent Technologies) and raw signal intensities were extracted with Feature Extraction v10.1 software (Agilent Technologies).

The dataset was normalized using quantile normalization [35] with a filter to remove all probes where the max log_2_ signal across arrays was less than 5. No background subtraction was performed, and the median feature pixel intensity was used as the raw signal before normalization. The filter for low intensity probes was used to prevent the prevalence of so many low-intensity probes (which tend to have smaller variance) from underestimating global and per-gene estimates of variance. A one-way ANOVA linear model was fit to the comparison to estimate the mean M values and calculated FDR and p-value for each miRNA for the comparison of interest. Adjusted p-values were produced using standard methods [39]. All procedures were carried out using functions in the R package *limma* in Bioconductor [36], [37]. All data comply with MIAME standards for microarray experiments [38], and have been deposited in Gene Expression Omnibus (http://www.ncbi.nlm.nih.gov/geo/; Accession Number to be provided during review).

### Quantitative real-time PCR

For analysis of transcript expression, GFP^+^ hESCs were sorted by FACS at indicated time points. RNA was isolated and cDNA synthesized from ∼50,000 hEB-derived cells or undifferentiated hESCs using the Taqman Gene Expression Cells-to-CT kit (Ambion). cDNA was quantitated using a Nanodrop ND-1000 Spectrophotometer (Nanodrop Technologies, ND Software version 3.3.0). Linear pre-amplification of target sequences was accomplished using the Applied Biosystems PreAmp system. Relative expression was determined using the TaqMan Assay (Applied Biosystems) on an ABI 7300 Real-Time PCR system with the following primer pairs (ABI): Lin28 (Hs00702802_s1), GATA4 (Hs00171403_m1), Nkx2-5 (Hs00231763_m1), sarcolipin (Hs00161903_m1;Hs01888464_s1), MLC2v (Hs00166405_m1;Hs01125721_m1), αMHC (Hs00411908_m1), cTnT (Hs00165960_m1), α-fetoprotein (Hs00173490_m1), nestin (Hs00707120_s1), Cx43 (Hs00748445_s1), Cx40 (Hs00979198_m1), Cx45 (Hs00271416_s1), and GAPDH (4326317E). Cycle times to detection were normalized against a reference gene, GAPDH, and relative changes were calculated using ABI Version 1.4 Sequence Detection Software.

For analysis of miRNA expression, miRNAs were isolated from hESCs or sorted cells using the mirVana miRNA Isolation kit (Ambion), and cDNA was reverse transcribed using the TaqMan MicroRNA RT kit (ABI). Following linear pre-amplification of miRNA sequences using the Applied Biosystems PreAmp system, relative expression was determined using singleplex TaqMan Assays (Applied Biosystems) with primer sets for human miR-125b (Applied Biosystems; 000449) and let-7d (Applied Biosystems; 4427975). Cycle times to detection were normalized against two reference sequences, RNU44 (001094) and RNU48 (001006), and relative changes were calculated as described.

### Overexpression and knockdown of miR-125b and let-7d

Pre-miR-125b precursor (Invitrogen/Ambion; PM10148), anti-miR-125b inhibitor (Invitrogen/Ambion; AM10148), and anti-let-7d inhibitor (Invitrogen/Ambion; AM1178) were transfected into undifferentiated hESCs using DharmaFECT Duo reagent (Dharmacon) according to the manufacturer's instructions. Initial dose-response experiments were performed with 15, 30, and 45 nM pre- and anti-miR reagent, as shown in **Figure 2C**. Once the optimum dose of 30 nM was established, all subsequent experiments were performed with 30 nM pre- or anti-miR reagent. For experiments with undifferentiated hESCs, cells were allowed to recover for 2 days before analysis. For experiments with differentiating hESCs, cells were re-transfected every 2 days until analysis.

### Luciferase reporter assay

Luciferase assays were performed in whole cell lysates using the dual luciferase reporter assay system (Promega) as described previously [40]. pRL-TK (Promega) encoding constitutively expressed *Renilla reniformis* luciferase was included in each transfection to normalize for transfection efficiency. At the time of pre-miR-125b and anti-miR-125b transfection, hESCs were co-transfected with 0.2 µg pMiR-125b-Luc reporter plasmid (Signosis; LR-0020) together with 0.1 µg of pRL-TK. pMiR-125b-Luc expresses *Photinus pyralis* luciferase under control of a CMV promoter, regulated by the presence of a miR-125b binding site within the 3′ UTR of the luciferase coding sequence. After 48 hrs, cell lysates were assayed for *Photinus* and *Renilla* luciferase activities. *Photinus* luciferase activity was normalized against *Renilla* luciferase activity and expressed as relative light units. Assays were performed in triplicate and repeated at least three times.

### Quantitative near-infrared fluorescence immunoblot analyis

Protein expression was determined by near-infrared fluorescence immunoblot analysis using a LI-COR Odyssey CLx (LI-COR Biosciences, Lincoln, NE) with a >4 log dynamic range. Briefly, whole cell lysates were separated on sodium dodecyl sulfate (SDS)–polyacrylamide gels (10%), then transferred in Tris-glycine, 20% methanol, 0.01% SDS onto PVDF membrane (Immobilon; Millipore) as previously described [41]. Membranes were blocked with phosphate-buffered saline-based Odyssey blocking buffer (927-40100; LI-COR), incubated with primary antibody at 1∶500 dilution in blocking buffer, then infrared dye-linked secondary antibody at 1∶20,000 dilution in blocking buffer. Primary antibodies included polyclonal rabbit anti-human Lin28 (H-44) (sc-67266; Santa Cruz Biotechnology) and goat anti-human actin (I-19) (sc-1616; Santa Cruz Biotechnology). Secondary antibody consisted of IRDye 680LT-conjugated goat anti-rabbit IgG (H+L) (827-11081; LI-COR). Bound antibodies were detected and quantitated with Odyssey v 3.0 software (LI-COR Biosciences, Lincoln, NE).

### Statistical analysis

For comparison of quantitative real-time PCR and immunoblot quantitation data, analysis of variance (ANOVA) with Fisher's post-hoc test was used. Where ANOVA indicated significant differences among groups, multiple comparisons were made using Student's *t*- test with Bonferroni correction. A p-value less than 0.05 was considered significant.

## Supporting Information

Figure S1 **Validation of gene expression during cardiomyocyte differentiation.** qPCR analysis of sorted αMHC-GFP^+^ and αMHC-GFP^−^ single cell suspensions from 14 day hEBs demonstrated upregulation of the cardiac-specific genes myosin light chain-2 ventricular (MLC2v), cardiac troponin I (cTnI), myocyte-specific/MADS box transcription enhancer factor 2C (Mef2c), GATA4, cyclin-dependent kinase inhibitor p21^Cip1^, and stem cell factor/c-kit ligand (SCF), and downregulation of the pluripotency factor, Nanog, as well as ectoderm-specific βIII-tubulin (βIII-tub) and the primitive endoderm marker, α-fetoprotein (AFP) in αMHC-GFP^+^ compared to αMHC-GFP^−^ cells. Data shown represent mean±s.e.m. (N = 5).
(TIF)

Figure S2 **miR-125b expression is similar between differentiating H7 and H9 hESCs.** Relative expression of endogenous miR-125b in undifferentiated, wild type H7 and H9 hESCs (Undiff) and wild type H7 and H9 hESCs grown in differentiation medium for 2, 3, 4, 8, and 14 days was assessed by qPCR. Similar expression patterns were seen over the course of differentiation for both lines *(top)*. Nanog expression was analyzed in parallel as an inverse measure of hESC differentiation *(bottom)*. Although miR-125b expression appears to be downregulated with differentiation of unselected hESC populations as shown here, it is specifically upregulated in differentiating CMs as shown in **Figure 2A**, where 8 and 14 day samples contain selected αMHC-GFP^+^ myocardial cells. This supports a mesoderm- and CM-specific role for miR-125b. Data shown are mean±s.e.m. (N = 4).
(TIF)

Table S1 **Conserved human miR-125b targets with aggregate probability of conserved targeting (P_CT_) >0.95.**
(DOCX)

Table S2 **Conserved human miR-125b targets with total context score ≤−0.45.**
(DOCX)
