# Supplementary figures and images for: miR-125b Promotes Early Germ Layer Specification through Lin28/let-7d and Preferential Differentiation of Mesoderm in Human Embryonic Stem Cells
Source: PLoS One. 2012 Apr 24;7(4):e36121. doi: 10.1371/journal.pone.0036121 (PMC3335794; doi:10.1371/journal.pone.0036121)

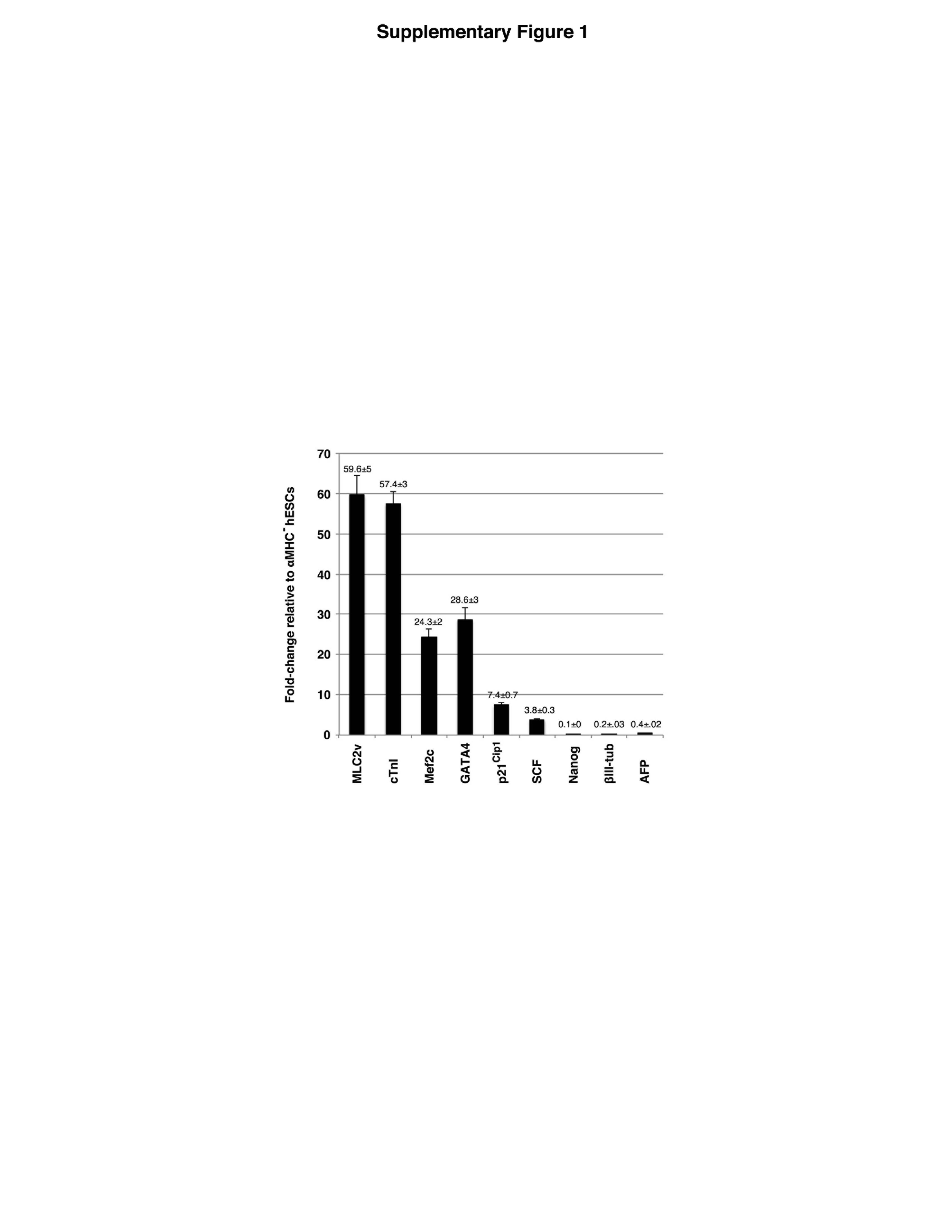

Supplement: Figure S1 — Validation of gene expression during cardiomyocyte differentiation. qPCR analysis of sorted αMHC-GFP+ and αMHC-GFP− single cell suspensions from 14 day hEBs demonstrated upregulation of the cardiac-specific genes myosin light chain-2 ventricular (MLC2v), cardiac troponin I (cTnI), myocyte-specific/MADS box transcription enhancer factor 2C (Mef2c), GATA4, cyclin-dependent kinase inhibitor p21Cip1, and stem cell factor/c-kit ligand (SCF), and downregulation of the pluripotency factor, Nanog, as well as ectoderm-specific βIII-tubulin (βIII-tub) and the primitive endoderm marker, α-fetoprotein (AFP) in αMHC-GFP+ compared to αMHC-GFP− cells. Data shown represent mean±s.e.m. (N = 5). (TIF) [file pone.0036121.s001.tif]

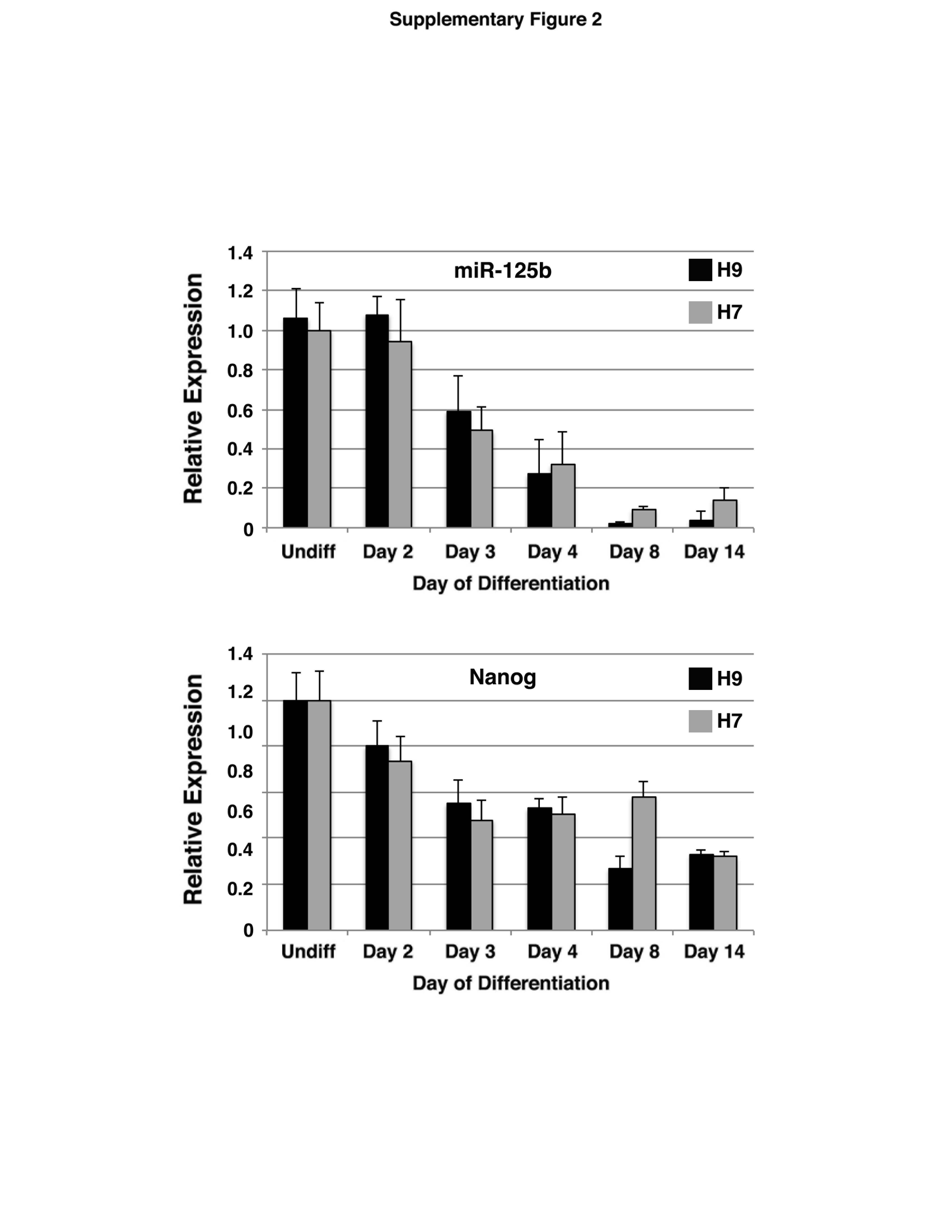

Supplement: Figure S2 — miR-125b expression is similar between differentiating H7 and H9 hESCs. Relative expression of endogenous miR-125b in undifferentiated, wild type H7 and H9 hESCs (Undiff) and wild type H7 and H9 hESCs grown in differentiation medium for 2, 3, 4, 8, and 14 days was assessed by qPCR. Similar expression patterns were seen over the course of differentiation for both lines (top). Nanog expression was analyzed in parallel as an inverse measure of hESC differentiation (bottom). Although miR-125b expression appears to be downregulated with differentiation of unselected hESC populations as shown here, it is specifically upregulated in differentiating CMs as shown in Figure 2A , where 8 and 14 day samples contain selected αMHC-GFP+ myocardial cells. This supports a mesoderm- and CM-specific role for miR-125b. Data shown are mean±s.e.m. (N = 4). (TIF) [file pone.0036121.s002.tif]
