# Supplementary material for: miR-125b Promotes Early Germ Layer Specification through Lin28/let-7d and Preferential Differentiation of Mesoderm in Human Embryonic Stem Cells
Source: PLoS One. 2012 Apr 24;7(4):e36121. doi: 10.1371/journal.pone.0036121 (PMC3335794; doi:10.1371/journal.pone.0036121)
Supplement: Table S1 — Conserved human miR-125b targets with aggregate probability of conserved targeting (PCT) >0.95. (DOCX) [file pone.0036121.s003.docx]

**Table S1. Conserved human miR-125b targets with aggregate probability of conserved targeting (P_CT_) >0.95**

| **Target Gene** | **Gene Name** | **Aggregate P_CT_** |
| --- | --- | --- |
| GCNT1 | glucosaminyl (N-acetyl) transferase 1, core 2 | > 0.99 |
| TMEM86A | transmembrane protein 86A | > 0.99 |
| PODXL | podocalyxin-like | > 0.99 |
| ARID3B | AT rich interactive domain 3B | > 0.99 |
| FLJ20309 | hypothetical protein FLJ20309 | 0.99 |
| SH3TC2 | SH3 domain and tetratricopeptide repeats 2 | 0.99 |
| PHF15 | PHD finger protein 15 | 0.98 |
| GJC1 | gap junction protein, gamma 1 | 0.98 |
| KLF13 | Kruppel-like factor 13 | 0.97 |
| OLFML2A | olfactomedin-like 2A | 0.97 |
| MFHAS1 | malignant fibrous histiocytoma amplified sequence 1 | 0.97 |
| IRF4 | interferon regulatory factor 4 | 0.97 |
| RAPGEF5 | Rap guanine nucleotide exchange factor 5 | 0.97 |
| LBH | limb bud and heart development homolog | 0.97 |
| ENPP1 | ectonucleotide pyrophosphatase/phosphodiesterase 1 | 0.97 |
| UBN1 | ubinuclein 1 | 0.97 |
| FAM176A | family with sequence similarity 176, member A | 0.97 |
| MXD4 | MAX dimerization protein 4 | 0.97 |
| SMURF1 | SMAD specific E3 ubiquitin protein ligase 1 | 0.97 |
| TLE3 | transducin-like enhancer of split 3 homolog | 0.97 |
| TRPS1 | trichorhinophalangeal syndrome I | 0.97 |
| KIAA1522 | KIAA1522 | 0.97 |
| ASAH3L | N-acylsphingosine amidohydrolase 3-like | 0.97 |
| UBE2R2 | ubiquitin-conjugating enzyme E2R 2 | 0.97 |
| SEMA4D | semaphorin 4D | 0.97 |
| LIN28 | lin-28 homolog | 0.97 |
| CPSF6 | cleavage and polyadenylation specific factor 6 | 0.97 |
| TGOLN2 | trans-golgi network protein 2 | 0.97 |
| OSBPL9 | oxysterol binding protein-like 9 | 0.97 |
| TBC1D1 | TBC1 domain family, member 1 | 0.97 |
| SLC39A9 | solute carrier family 39, member 9 | 0.96 |
| ENPEP | glutamyl aminopeptidase | 0.96 |
| ST8SIA4 | ST8 alpha-N-acetyl-neuraminide alpha-2,8-sialyltransferase 4 | 0.96 |
| GRB10 | growth factor receptor-bound protein 10 | 0.96 |
| MYT1 | myelin transcription factor 1 | 0.96 |
| STARD13 | StAR-related lipid transfer domain containing 13 | 0.96 |
| PTAR1 | protein prenyltransferase alpha subunit repeat containing 1 | 0.96 |
| BMF | Bcl2 modifying factor | 0.96 |
| ZNRF3 | zinc and ring finger 3 | 0.96 |
| ZSCAN29 | zinc finger and SCAN domain containing 29 | 0.96 |
| SPTB | erythrocytic beta-spectrin | 0.96 |
| NIN | ninein | 0.96 |
| SMG1 | PI-3-kinase-related kinase SMG-1 | 0.96 |
| NUP210 | nucleoporin 210 | 0.96 |
| GGA2 | golgi associated, gamma adaptinARF binding protein 2 | 0.96 |
| DUS1L | dihydrouridine synthase 1-like | 0.96 |
| MTF1 | metal-regulatory transcription factor 1 | 0.96 |
| C14orf43 | chromosome 14 open reading frame 43 | 0.96 |
| PPAT | phosphoribosyl pyrophosphate amidotransferase | 0.96 |
| SMEK1 | suppressor of mek1 homolog 1 | 0.96 |
| ORC2L | origin recognition complex, subunit 2-like | 0.96 |
| ACHE | acetylcholinesterase | 0.96 |
