# Supplementary material for: miR-125b Promotes Early Germ Layer Specification through Lin28/let-7d and Preferential Differentiation of Mesoderm in Human Embryonic Stem Cells
Source: PLoS One. 2012 Apr 24;7(4):e36121. doi: 10.1371/journal.pone.0036121 (PMC3335794; doi:10.1371/journal.pone.0036121)
Supplement: Table S2 — Conserved human miR-125b targets with total context score ≤−0.45. (DOCX) [file pone.0036121.s004.docx]

**Table S2. Conserved human miR-125b targets with total context score ≤ -0.45**

| **Target Gene** | **Gene Name** | **Total Context Score** |
| --- | --- | --- |
| STARD13 | StAR-related lipid transfer domain containing 13 | -0.99 |
| ZNF792 | zinc finger protein 792 | -0.73 |
| SH3TC2 | SH3 domain and tetratricopeptide repeats 2 | -0.73 |
| GCNT1 | glucosaminyl (N-acetyl) transferase 1, core 2 | -0.67 |
| FUT4 | fucosyltransferase 4 | -0.65 |
| NAIF1 | nuclear apoptosis inducing factor 1 | -0.62 |
| SMEK1 | suppressor of mek1 homolog 1 | -0.61 |
| IER3IP1 | immediate early response 3 interacting protein 1 | -0.60 |
| ZSCAN29 | zinc finger and SCAN domain containing 29 | -0.58 |
| PRRC1 | proline-rich coiled-coil 1 | -0.58 |
| C10orf104 | chromosome 10 open reading frame 104 | -0.58 |
| IRF4 | interferon regulatory factor 4 | -0.58 |
| ACHE | acetylcholinesterase | -0.57 |
| BAK1 | BCL2-antagonist/killer 1 | -0.56 |
| VPS4B | vacuolar protein sorting 4 homolog B | -0.56 |
| LACTB | Beta-lactamase | -0.56 |
| COL4A3 | collagen, type IV, alpha 3 | -0.56 |
| NPL | N-acetylneuraminate pyruvate lyase | -0.56 |
| NUP210 | nucleoporin 210kDa | -0.55 |
| TMEM168 | transmembrane protein 168 | -0.53 |
| BAP1 | BRCA1 associated protein-1 | -0.52 |
| MFHAS1 | malignant fibrous histiocytoma amplified sequence 1 | -0.52 |
| ENPEP | glutamyl aminopeptidase A | -0.52 |
| TRIM71 | tripartite motif-containing 71 | -0.51 |
| ARID3B | AT rich interactive domain 3B | -0.51 |
| ZC3H7B | zinc finger CCCH-type containing 7B | -0.51 |
| MEGF9 | multiple EGF-like-domains 9 | -0.51 |
| KLF13 | Kruppel-like factor 13 | -0.51 |
| ICHTHYIN | ichthyin protein | -0.51 |
| TMEM77 | transmembrane protein 77 | -0.50 |
| TNFSF4 | tumor necrosis factor (ligand) superfamily, member 4 | -0.49 |
| OLFML2A | olfactomedin-like 2A | -0.49 |
| PCTP | phosphatidylcholine transfer protein | -0.49 |
| ANKRD42 | ankyrin repeat domain 42 | -0.48 |
| ENTPD1 | ectonucleoside triphosphate diphosphohydrolase 1 | -0.48 |
| DUS1L | dihydrouridine synthase 1-like | -0.48 |
| LFNG | O-fucosylpeptide 3-beta-N-acetylglucosaminyltransferase | -0.48 |
| TTC7A | tetratricopeptide repeat domain 7A | -0.48 |
| PODXL | podocalyxin-like | -0.47 |
| SLC39A9 | solute carrier family 39, member 9 | -0.47 |
| BIN2 | bridging integrator 2 | -0.47 |
| SLITRK6 | SLIT and NTRK-like family, member 6 | -0.46 |
| GANC | neutral alpha-glucosidase C | -0.46 |
| CCNJ | cyclin J | -0.46 |
| PTPN18 | non-receptor protein tyrosine phosphatase, type 18 | -0.46 |
| OSBPL9 | oxysterol binding protein-like 9 | -0.45 |
| VAX1 | ventral anterior homeobox 1 | -0.45 |
| PPP1R12B | protein phosphatase 1, subunit 12B | -0.45 |
